# Maternal immune activation and adolescent alcohol exposure increase alcohol drinking and disrupt cortical-striatal-hippocampal oscillations in adult offspring

**DOI:** 10.1038/s41398-022-02065-y

**Published:** 2022-07-20

**Authors:** Angela M. Henricks, Emily D. K. Sullivan, Lucas L. Dwiel, Judy Y. Li, Diana J. Wallin, Jibran Y. Khokhar, Wilder T. Doucette

**Affiliations:** 1 Department of Psychology, Washington State University, Pullman, WA US; 2 Department of Psychiatry, Geisel School of Medicine at Dartmouth, Hanover, NH US; 3 Department of Biomedical Sciences, University of Guelph, Guelph, ON Canada

**Keywords:** Addiction, Neuroscience

## Abstract

Maternal immune activation (MIA) is strongly associated with an increased risk of developing mental illness in adulthood, which often co-occurs with alcohol misuse. The current study aimed to begin to determine whether MIA, combined with adolescent alcohol exposure (AE), could be used as a model with which we could study the neurobiological mechanisms behind such co-occurring disorders. Pregnant Sprague-Dawley rats were treated with polyI:C or saline on gestational day 15. Half of the offspring were given continuous access to alcohol during adolescence, leading to four experimental groups: controls, MIA, AE, and Dual (MIA + AE). We then evaluated whether MIA and/or AE alter: (1) alcohol consumption; (2) locomotor behavior; and (3) cortical-striatal-hippocampal local field potentials (LFPs) in adult offspring. Dual rats, particularly females, drank significantly more alcohol in adulthood compared to all other groups. MIA led to reduced locomotor behavior in males only. Using machine learning to build predictive models from LFPs, we were able to differentiate Dual rats from control rats and AE rats in both sexes, and Dual rats from MIA rats in females. These data suggest that Dual “hits” (MIA + AE) increases substance use behavior and disrupts activity in reward-related circuits, and that this may be a valuable heuristic model we can use to study the neurobiological underpinnings of co-occurring disorders. Our future work aims to extend these findings to other addictive substances to enhance the translational relevance of this model, as well as determine whether amelioration of these circuit disruptions can reduce substance use behavior.

## Introduction

Approximately 9.5 million adults in the United States are living with both a substance use disorder (SUD) and mental illness [1], typically referred to as “co-occurring disorders.” Alcohol is one of the most commonly misused substances in this population, with approximately 32% of individuals with mental illness engage in problematic drinking [1]. Individuals with co-occurring disorders are less responsive to treatment and experience higher rates of relapse, homelessness, incarceration, and suicide compared to individuals with a single disorder [2]. One reason for this difference in morbidity is because co-occurring disorders are notoriously difficult to treat and require integrative care, and the available pharmacotherapies are largely ineffective [3, 4]. A major barrier to the development of better therapies is that there is still much to learn regarding the neurobiological mechanisms underlying these co-occurring disorders [5, 6].

From a neurobiological perspective, it has been consistently demonstrated that the brain is highly susceptible to the harmful effects of environmental stressors in the early stages of development. One major environmental risk factor is prenatal exposure to infection [7–10]. Systemic viral infections, like influenza or rubella, in pregnant women have been repeatedly associated with an increased incidence of psychosis- and mood-related disorders in offspring (e.g., schizophrenia, bipolar disorder, and depression) [8, 9], and these mental illnesses are often comorbid with alcohol misuse [1]. However, since not all individuals exposed to infection in the prenatal environment develop a mental illness, it is likely that prenatal stressors combined with a “second-hit” during other critical periods of development (e.g., adolescence) further increase the probability of developing a mental illness in adulthood [8–10]. There is evidence for this “two-hit” model, reviewed elsewhere [9, 10], indicating that a possible adolescent stressor is alcohol and/or drug use. Using maternal immune activation (MIA) to mimic prenatal exposure to infection in rodents, we tested the hypothesis that MIA combined with adolescent alcohol exposure (AE) might serve as a useful heuristic with which we can begin to study co-occurring disorders.

We further hypothesized that MIA and/or AE would disrupt neural circuit activity in regions that regulate reward-related behaviors. Cortical, striatal, and hippocampal circuits are all implicated in alcohol misuse [11]. Specifically, activity in the medial prefrontal cortex (mPFC) and nucleus accumbens (NAc) drive responses to rewarding substances like alcohol [11, 12], while the PFC and hippocampus appear particularly important in associative learning and coordinating responses to drug cues [12, 13]. Disruptions in these circuits are also seen in mental illness. Our previous clinical work has demonstrated that individuals with schizophrenia and co-occurring substance use disorder have reduced functional connectivity between the NAc and mPFC [14]. There is also a multitude of data showing structural and functional changes to the hippocampus in schizophrenia and bipolar disorder, which might be related to symptoms involving learning and regulating emotions [13, 15]. Others have also shown that MIA offspring have reduced connectivity between the mPFC and the CA1 of the hippocampus which correlates with abnormal prepulse inhibition, a well-characterized symptom of MIA exposure consistent with the sensorimotor gating deficits observed in schizophrenia and bipolar disorder [16, 17]. We therefore hypothesize that if these regions contain information regarding alcohol drinking in MIA rats, they may also serve as therapeutic targets in future research [18].

The current set of experiments investigated the impact of MIA, AE, and MIA + AE (Dual) on alcohol drinking behavior and local field potentials (LFPs) recorded from the mPFC, NAc shell, and CA1 in male and female rats. LFPs represent aggregated electrical signals from neurons that appear to largely reflect synchronized synaptic inputs, and are thus useful for understanding how information flows through neural circuits [19]. LFPs are also a translationally-relevant method to measure activity within and connectivity between brain regions in freely behaving animals [20]. We, therefore, aimed to determine whether: (1) MIA and/or AE alters alcohol consumption; (2) MIA and/or AE alters locomotor behavior; and (3) cortical-striatal-hippocampal LFPs could predict MIA and/or AE exposure.

## Materials and methods

### General experimental design

Experiment 1 allowed us to evaluate the impact of MIA and AE on adulthood alcohol drinking behavior. Male and female offspring were divided into four groups: control, MIA, AE, or Dual. Since MIA rats have shown both hypo- and hyperactive locomotor behavior in previous studies [21–23], we also tested rats’ locomotor response to a novel environment in adulthood to verify that our procedures caused a known behavioral phenotype of MIA. Rats were then trained to drink alcohol in their home cage, as described below.

**Fig. 1:**
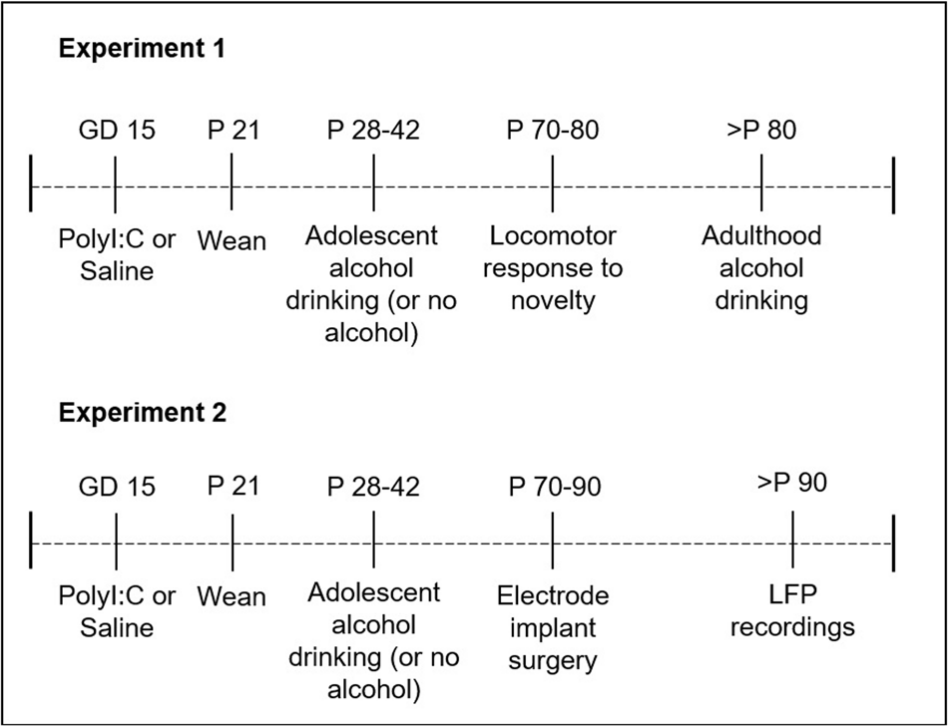
Experimental timelines. Dams were exposed to polyI:C or saline on gestational day (GD) 15. Half of the offspring were then exposed to continuous alcohol from postnatal day (P) 28-42. Behavioral and electrophysiological experiments began in adulthood (>P 70).

Experiment 2 allowed us to evaluate the impact of MIA and AE on cortical-striatal-hippocampal oscillations. A separate group of adult male and female control, MIA, AE, or Dual rats were implanted with electrodes targeting the prelimbic (PL) and infralimbic (IL) mPFC, NAc shell, and CA1. Following recovery, each rat underwent two, 30-minute recording sessions to measure baseline neural circuit activity, and we used an unbiased machine-learning approach to determine whether cortical-striatal-hippocampal LFPs could predict MIA and/or AE exposure. See Fig. 1 for the experimental timelines.

### Animals

Timed-pregnant Sprague-Dawley rats were ordered to arrive on a gestational day (GD) 8 (Charles River), and allowed to acclimate to the housing environment for 7 days prior to experimentation. Dams were housed individually on a reverse 12-hour light cycle with *ad libitum* access to food and water. Pups were weaned on postnatal day (P) 21 and housed in same-sex pairs until P 28, after which all animals were individually housed. All experiments were carried out in accordance with the National Institute of Health Guide for the Care and Use of Laboratory Animals (NIH Publications No. 80–23) and were approved by the Institutional Animal Care and Use Committee of Dartmouth College.

### Maternal immune activation

Pregnant dams were randomly assigned to receive polyinosinic:polycytidylic acid [polyI:C, 4 mg/kg, IV] (Tocris Bioscience) or saline (1 mL/kg, IV) on GD 15. PolyI:C is a synthetic analog of double-stranded RNA that leads to a heightened immune response in rats [10]. Dams’ body weight, food, and water intake were monitored at −24, 0, 24, and 48 hours from the injection. Blood was collected 2 hours after injection to measure the pro-inflammatory cytokines IL6 and TNFα. Both male and female pups were allowed to develop normally and were weaned on P 21.

### Cytokine ELISA

Serum samples from dams were analyzed via enzyme-linked immunosorbent assay (ELISA) for IL6 and TNFα (Thermo Fisher Scientific) following the manufacturer’s recommendations.

### Adolescent alcohol exposure

AE and Dual rats were allowed to drink 10% alcohol (v/v) in their home cage from P 28–42, consistent with our previous work [24]. Rats were given 24 hour access to alcohol and water, and the weight of each bottle was measured daily. The position of each bottle was rotated daily to avoid positional preference, and rats were weighed weekly to calculate weight-adjusted alcohol consumption (g/kg).

### Locomotor response to a novel environment

On approximately P 70, rats were allowed to explore a novel open field (60 cm × 60 cm × 33 cm) in the dark for 25 minutes. Infrared cameras recorded the behavior, which was analyzed with EthoVision behavioral tracking software (Noldus Information Technology). Total distance traveled, frequency in the center zone, and time in the center zone was calculated and used for data analyses.

### Adulthood alcohol drinking

On approximately P 80, rats were trained to drink 10% alcohol (v/v) in their home cage for 90 min/day, 5 days/week using a sucrose fade technique like that described previously [25]. Briefly, rats were allowed to drink 5% sucrose in water during week 1, then 5% sucrose + 10% alcohol during week 2, then 2.5% sucrose + 10% alcohol during week 3, then only 10% alcohol in water for three more weeks. Rats were weighed weekly to assess the amount of alcohol consumed in g/kg.

### Surgery

Electrodes were designed and constructed in-house and were similar to those used in our previous publications [26–28]. Animals were anesthetized with isoflurane gas (4% induction, 2% maintenance) and mounted in a stereotaxic frame. Custom electrodes were implanted bilaterally targeting the PL mPFC (from bregma: DV −4 mm; AP +3.4 mm; ML ±0.75 mm), IL mPFC (from bregma: DV −5 mm; AP +3.4 mm; ML ±0.75 mm), NAc shell (from bregma: DV −8 mm; AP +1.2 mm; ML ±1.0 mm), and CA1 of the hippocampus (from bregma: DV −2.5 mm; AP −3.8 mm; ML ±2.5 mm). Four stainless steel skull screws were placed around the electrode site and dental cement (Dentsply) was applied to secure the electrodes in place. Rats were allowed to recover for at least 7 days before any experimentation began.

#### Histology

At the end of the experiments, rats were euthanized via CO_2_ gas inhalation. Brains were harvested from rats implanted with electrodes and flash frozen in 2-methylbutane on dry ice. The tissue was stored at −20 °C prior to being sectioned at 50 μm using a Leica CM1850 cryostat and stained with thionin. Electrode placement was verified using a Leica A60 microscope. Out of the 84 rats implanted, we were unable to check complete histology on 8 brains due to tissue damage that occurred during the collection process: 3 control, 1 MIA, 2 AE, and 2 Dual brains. The LFP data for these rats were excluded from analyses. Figure 5A depicts representations of electrode placements.

### Local field potential recordings

LFPs were recorded from each awake, freely behaving rat in a standard operant chamber (MedAssociates). Rats were allowed to move about the chamber, but there was no task involved and rats did not have any prior experience in the chambers. Data from each recording were analyzed using frequency ranges from the rodent literature (*δ* = 1–4 Hz, *θ* = 5–10 Hz, *α* = 11–14 Hz, *β* = 15–30 Hz, low γ = 45–65 Hz, and high *γ* = 70–90 Hz). LFP signal processing to characterize the power spectral densities (PSD) within, and coherence between brain regions, for each rat was calculated using custom code written for Matlab, as we have previously published [26–28].

### Statistical analysis

#### Dam analysis

Pro-inflammatory cytokine levels were analyzed using independent sample *t* tests comparing Control and MIA dams. A repeated measures ANOVA was used to compare behavioral and physiological responses to polyI:C using time as the within-subject variable and group (Control or MIA) as the between-subject variable.

#### Behavioral analysis in offspring

The average g/kg of alcohol consumed in adolescence and adulthood was analyzed using a repeated measures ANOVA, with time as the within-subject variable and MIA, AE, and sex as the between-subject variables. The average total distance traveled, frequency in the center, and time in the center was calculated for the entire locomotor response to the novelty session. A three-way ANOVA analyzed the impact of MIA, AE, and sex. All behavioral data were analyzed with dam number as a covariate to control for any possible litter effects. Based on our previous findings [24], we estimated that 8–12 animals/group/sex would yield sufficient power to determine whether MIA and/or AE impacted alcohol consumption in adulthood. Researchers were not blinded to the experimental conditions.

#### LFP oscillation analysis

Similar to our previous publications [26–28] we built general linear models to classify rats based on group assignment (i.e., control, MIA, AE, or Dual) using LFP oscillation data. Since each rat underwent two baseline recording sessions, we used data from both sessions to build baseline models. Data were then calculated in 5 second bins, with each bin representing one sample in the models. Using a “leave-one-out” (LOO) approach, models were then trained on all data minus one animal from each group, and then the model was tested on the left-out animal. To account for overrepresenting animals with more “clean” data (i.e., low noise) than other animals with less “clean” data (i.e., high noise), each model used only 1200 samples from each animal. All possible combinations of LOO were analyzed, and each LOO combination was run 100 times to account for sub-sampling. Model performance is reported as the mean area under the receiver operating characteristic curve (AUC) ± 95% confidence interval. The relevant code used to create these models is available on Github: https://github.com/lucasdwi/code/blob/greenlab/notes/angelaMIANotes.m.

Because we used multiple recording sessions from the same animal and 5-second bins as samples, we also evaluated models built on permutations of binary rat groupings (“animal detector”), as previously described [28]. This was done by keeping the LFP oscillation data together with the rat it was recorded from, but then shuffling the group assignment of each rat’s set of recordings. The “animal detector” test thus allowed us to determine how much of our model accuracy was simply due to the ability of the algorithm to predict individual differences in oscillations *not* related to group assignment. We then compared the model performance of the real data to the “animal detector.” If the real model performed with greater accuracy than the “animal detector,” it indicated that information existed in the LFP signal regarding group assignment. We then implemented exhaustive single-feature regressions using each LFP predictor to determine the relative information content of each neural feature. Figure 5B depicts the model building approach.

## Results

### The Impact of PolyI:C on dams

**Fig. 2:**
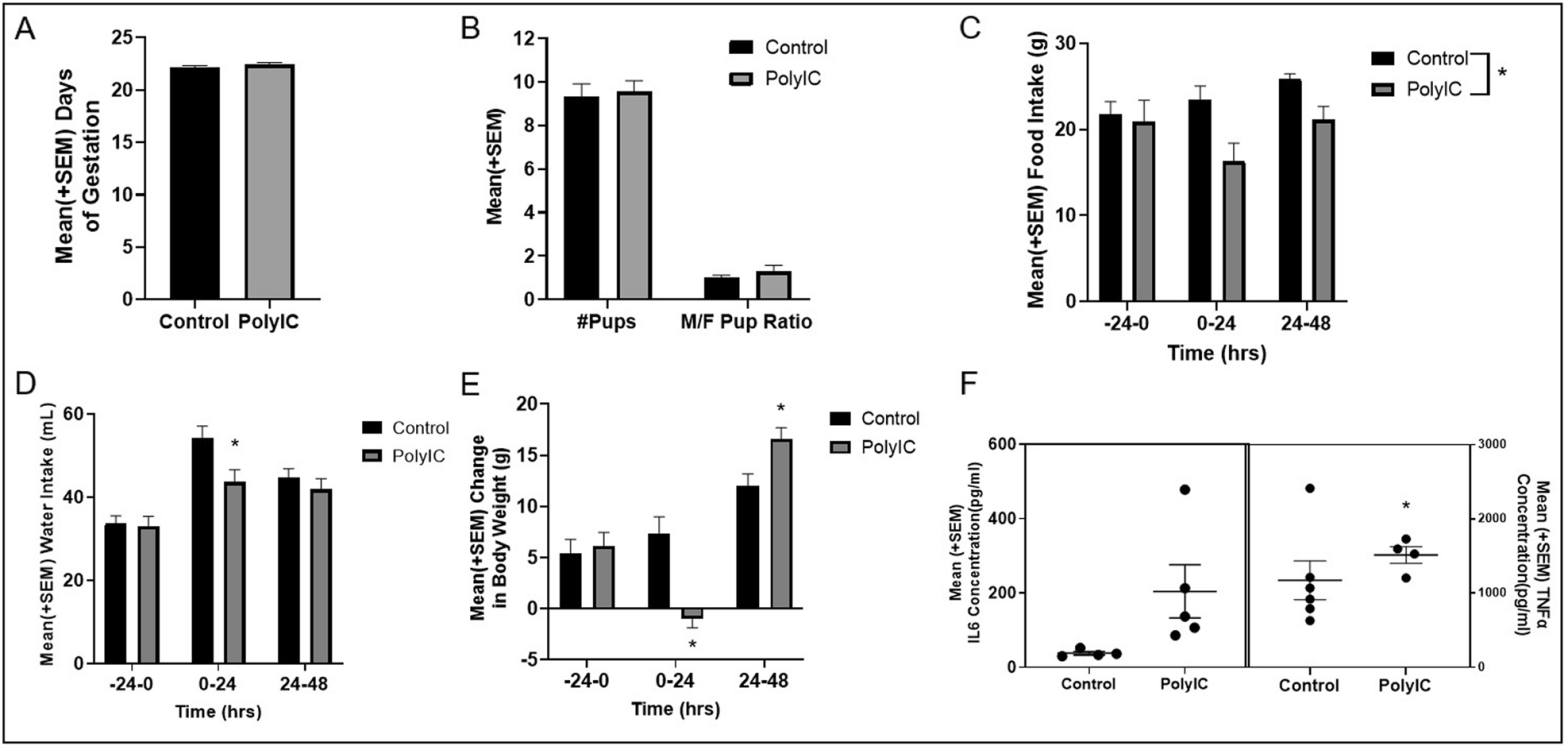
The impact if polyI:C on dams. **A** PolyI:C did not impact the number of days of gestation compared to saline-treated dams (controls) (*p* > 0.05; *n* = 9/group). **B** PolyI:C did not impact the number of pups born or M/F pup ratio compared to control dams (*p* > 0.05; *n* = 9/group). **C** Dams treated with polyI:C ate less food than control dams overall (*p* = 0.002; *n* = 9/group). **D** Dams treated with polyI:C drank less water than control dams 24 hours after injection (*p* < 0.05; *n* = 8/group). **E** PolyI:C caused a reduction in body weight 24 hours after injection, but an increase 48 hours after injection, compared to control dams (*p* < 0.05; *n* = 9/group). **F** The im*p*act of polyI:C on IL6 and TNFα concentration 2 hours post-injection. Each dot represents an individual dam. PolyI:C significantly enhanced TNFα levels (*p* = 0.006; *n* = 4–5/group), but not IL6 levels (*p* = 0.08; *n* = 4–5/group).

Two-way ANOVAs showed that polyI:C did not influence the total number of days in gestation, the number of pups born, or the M/F pup ratio (all *p* values > 0.05; *n* = 9/group; Fig. 2A, B). However, polyI:C significantly reduced food intake compared to control dams [*F*(1,16) = 13.32, *p* = 0.002; *n* = 9/group; Fig. 2C]. There was also a significant time*group interaction for water intake [*F*(2,28) = 4.60, *p* = 0.02; *n* = 8/group; Fig. 2D], and weight gain [*F*(2,32) = 12.69, *p* < 0.001; *n* = 9/group; Fig. 2E], with post-hoc tests showing that polyI:C dams drank less water and gained less weight than control dams for the first 24 hours post injection (*p’s* < 0.05). PolyI:C significantly enhanced circulating TNFα [*t*(7) = −3.89, *p* = 0.006], but not IL6 [*t*(4.04) = −2.31, *p* = 0.08] levels, two hours after injection (*n* = 4–5/group; Fig. 2F).

### Experiment 1: The impact of MIA and AE on behavior

#### Alcohol consumption in adolescence

**Fig. 3:**
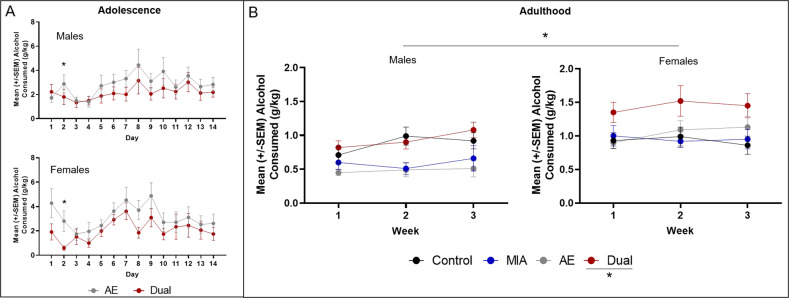
The impact of MIA and AE on offspring drinking behavior. **A** Average alcohol consumed (g/kg) during adolescence (P 28–42) in male and female offspring from dams exposed to polyI:C (Dual) or saline (AE). There were no significant differences between groups, except during session 2 (*p* > 0.05; *n* = 9–11/group/sex from 4 dams). **B** Average alcohol consumed (g/kg) in a limited access paradigm across 3 weeks in adult control, MIA, AE, and dual rats. Drinking rates increased across time in all groups (*p* = 0.05), but Dual offspring drank significantly more alcohol than all other groups during weeks 2 and 3 (*p* < 0.05; *n* = 8–11/group/sex from 12 dams). Female rats drank more alcohol than males (*p* < 0.05).

A repeated measures ANOVA revealed a significant effect of session [*F*(13, 442) = 2.26, *p* = 0.007, *n*^*2*^*p* = 0.06], a session*MIA interaction [*F*(13, 442) = 2.61, *p* = 0.002, *n*^*2*^*p* = 0.07], and a session*sex interaction [*F*(13, 442) = 2.83, *p* = 0.001, *n*^*2*^*p* = 0.08; *n* = 9–11/group/sex from four dams). Post-hoc analyses revealed a significant effect of MIA on drinking only during session 2 (*p* < 0.05), but did not reveal any sessions in which there was a significant difference in alcohol consumed between male and female rats (all *p* values >0.05; Fig. 3A).

#### Alcohol consumption in adulthood

See the supplemental materials for the sucrose fade data. For 10% alcohol alone, a repeated measures ANOVA revealed a significant effect of week [*F*(2, 134) = 5.57, *p* = 0.005, *n*^*2*^*p* = 0.08], a significant effect of sex [*F*(1, 67) = 19.02, *p* < 0.001, *n*^*2*^*p* = 0.22], a significant week*AE interaction [*F*(2, 134) = 4.64, *p* = 0.01, *n*^*2*^*p* = 0.07], a significant AE*sex interaction [*F*(1, 67) = 4.19, *p* = 0.045, *n*^*2*^*p* = 0.06], a significant MIA*AE interaction [*F*(1, 67) = 5.36, *p* = 0.02, *n*^*2*^*p* = 0.07], and a significant week*MIA*AE interaction [*F*(2, 134) = 4.22, *p* = 0.02, *n*^*2*^*p* = 0.06; *n* = 8–11/group/sex from 12 dams]. Post-hoc analyses revealed that: (1) across weeks, alcohol consumption increased in all groups; (2) Dual rats drank significantly more alcohol than Control, MIA, and AE rats in weeks 2 and 3 (with a trend for week 1, *p* = 0.059); and (3) female rats drank more alcohol overall compared to male rats (all *p* values < 0.05; Fig. 3B).

#### Locomotor response to novelty in adulthood

**Fig. 4:**
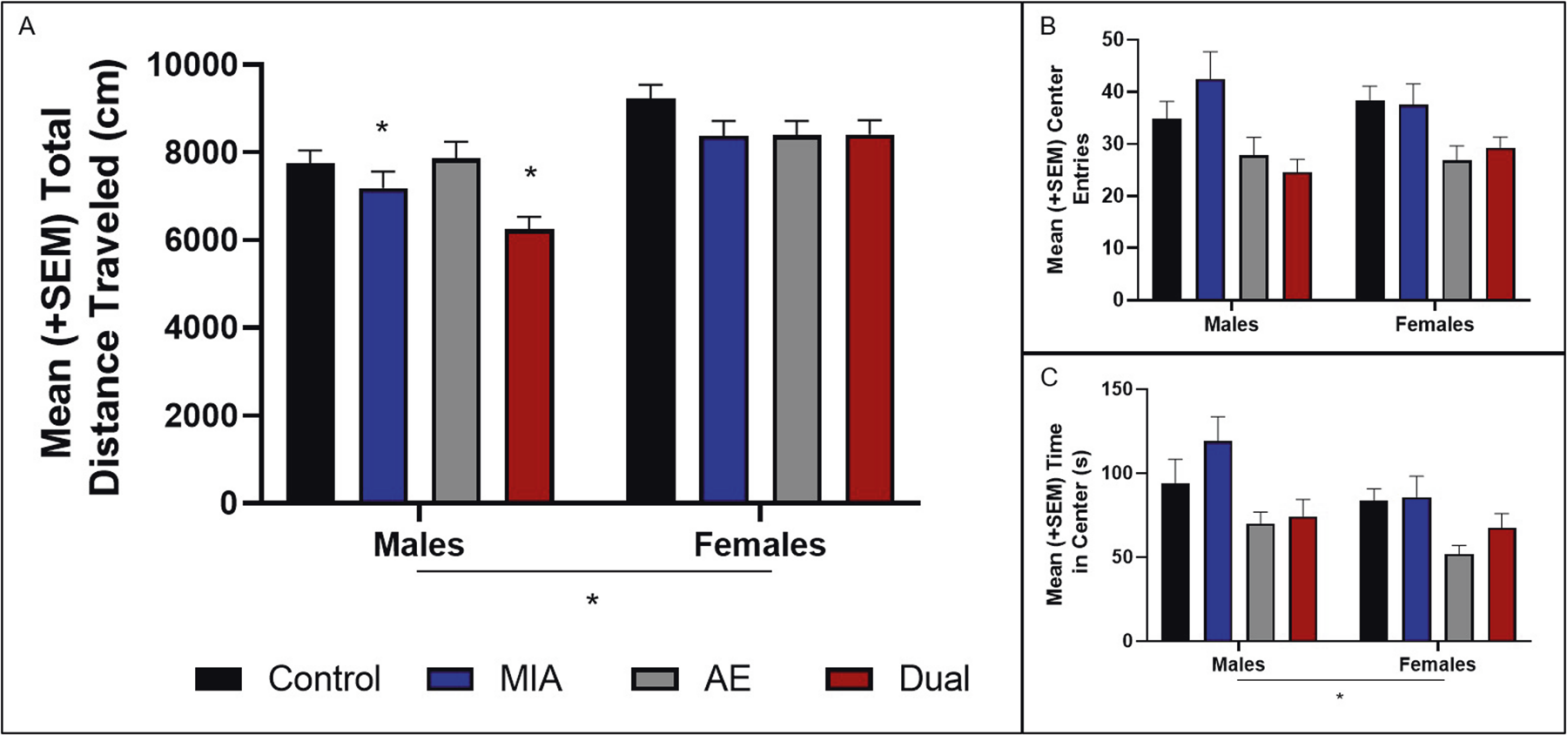
The impact of MIA and AE on offspring locomotor behavior. **A** MIA reduced total distance traveled in locomotor response to novelty task in male rats (*p* < 0.05). Female rats overall moved more than male rats (*p* < 0.05; *n* = 9–11/group/sex from 8 dams). **B** MIA and/or AE did not impact the number of center entries; or **C** the time spent in the center (*p* > 0.05). Overall, female rats spent less time in the center zone compared to male rats (*p* < 0.05).

A three-way ANOVA revealed a significant effect of sex [*F*(1, 72) = 32.50, *p* < 0.001, *n*^*2*^*p* = 0.31], MIA [*F*(1, 72) = 11.53, *p* = .001, *n*^*2*^*p* = 0.14], dam number [*F*(1, 72) = 4.69, *p* = 0.03, *n*^*2*^*p* = 0.06], and a sex*MIA*AE interaction [*F*(1, 72) = 4.86, *p* = 0.03, *n*^*2*^*p* = 0.06; *n* = 9–11/group/sex from eight dams). Post-hoc analyses revealed that male MIA and Dual rats moved less than Control and AE rats (*p* < 0.05), and female rats overall moved more than male rats (*p* < 0.05; Fig. 4A). There was also a significant effect of sex for time in the center [*F*(1, 72) = 5.06, *p* = 0.03, *n*^*2*^*p* = 0.07], with post-hoc analyses revealing that males overall spent more time in the center zone compared to females (*p* < 0.05). However, there were no other significant effects for center entries or time spent in the center zone (Fig. 4B, C).

### Experiment 2: The impact of MIA and AE on LFP oscillations

**Fig. 5:**
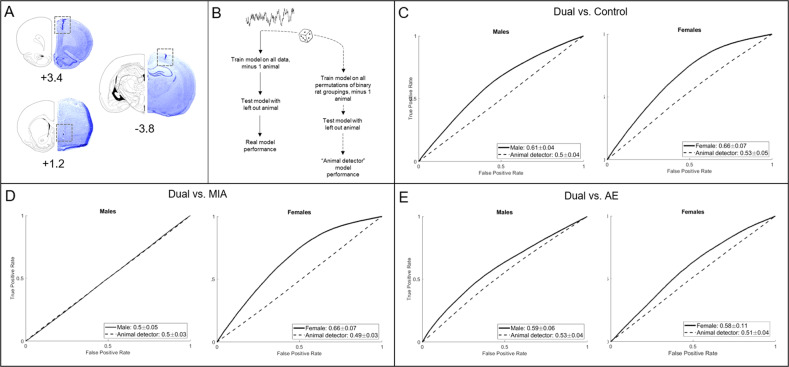
Cortical-striatal-hippocampal LFPs predict MIA and/or AE exposure. **A** Histologic representation of lesions caused by electrode cannula in the mPFC (+3.4 mm from bregma), NAcSh (+1.2 mm from bregma), and CA1 (−3.8 mm from bregma). Electrode wires extended 1 mm from the end of the cannula for the NAcSh, CA1, and PL, and 2 mm for the IL. **B** Schematic representation of the baseline model building. **C** LFPs predicted Dual rats from control rats better than the “animal detector” in both males and females (5–10/group/sex from 7 dams). **D** LFPs predicted Dual rats from MIA rats better than the “animal detector” in females, but not in males (*n* = 5–13/group from 6 dams). **E** LFPs predicted Dual rats from AE rats better than the “animal detector” in both males and females (*n* = 5–10/group/sex from four dams).

In order to identify how MIA and AE impact cortical-striatal-hippocampal LFP oscillations, we built predictive models comparing Duals to control, MIA, and AE groups individually. LFP data were analyzed for each sex separately. Because the Dual rats showed significant increases in alcohol drinking compared to other groups, we used Dual rats as the comparison group. Using LFPs to predict Dual rats from Control rats, models for each sex outperformed the “animal detector” (Males real mean accuracy = 0.61 ± 0.04; Females real mean accuracy: 0.66 ± 0.07; *n* = 5–10/group/sex from seven dams; Fig. 5C). Further, models were able to predict Dual vs. MIA rats in females (real mean accuracy = 0.66 ± 0.07; *n* = 5–13/group from six dams), but not in males (real mean accuracy = 0.50 ± 0.05; *n* = 10–12/group from seven dams; Fig. 5D), and Dual vs. AE rats in both sexes (Males real mean accuracy = 0.59 ± 0.06; Females real mean accuracy: 0.58 ± 0.11; *n* = 5–10/group/sex from four dams; Fig. 5E). The “animal detector” models estimated chance predictions in all cases, with a mean accuracy ranging from 0.49 ± 0.03–0.53 ± 0.05 (Fig. 5C–E). Representative raw LFPs, a PSD, and a coherence plot can be found in the supplementary materials.

Based on single-feature regression analyses, Table 1 shows the five neural features containing the most information (i.e., with the highest individual prediction accuracies) for each of the models that performed above chance estimates. It is interesting to note that most of the predictive individual features are power features.

**Table 1:** The neural features containing the most information for each of the models that performed above chance estimates.

Dual vs. Control		Dual vs. MIA		Dual vs. AE	
Male	Female	Male	Female	Male	Female
IL_L_ – CA1_R_ α	IL_R_ lγ	----	IL_R_ β	CA1_R_ β	CA1_L_ β
CA1_R_ lγ	IL_R_ δ	----	IL_R_ lγ	PL_L_ δ	NAc_L_ β
PL_R_ α	IL_R_ θ	----	IL_R_ hγ	PL_R_ δ	IL_R_ β
NAc_L_ δ	IL_L_ δ	----	PL_L_ β	PL_L_ θ	PL_L_ β
IL_L_ – CA1_L_ α	IL_L_ lγ	----	IL_L_ β	CA1_L_ β	IL_R_ lγ

The 5 LFP features with the highest individual predictive accuracies of Dual vs. Control, Dual vs. MIA in females, and Dual vs. AE. Frequency bands [delta (δ), theta (θ), alpha (α), beta (β), low gamma (lγ), and high gamma (hγ)] are described for power features within, and coherence between, neural sites (*NAc* nucleus accumbens shell, *CA1* dorsal hippocampus, *PL* prelimbic mPFC, *IL* infralimbic mPFC). Left or right hemisphere is depicted as a subscript.

## Discussion

MIA is a relatively well-characterized heuristic model in terms of the behavioral similarities to mental illnesses like schizophrenia, bipolar disorder, and depression [8]; MIA leads to maladaptive changes in locomotor behavior, affect, cognitive flexibility, sensorimotor gating, and social interactions [9, 10]. However, even though mental illness often co-occurs with substance misuse, the current set of experiments is one of the very few studies (discussed below) to test whether MIA rats might be more prone to addiction-like behaviors, and are thus a significant addition to the literature. Our data specifically indicate that MIA or AE alone does not impact alcohol drinking behavior, but that “two-hits” (e.g., MIA + AE; Dual) leads to enhanced alcohol consumption in adult offspring, particularly in females. Further, predictive models using cortical-striatal-hippocampal LFPs can differentiate Dual rats from controls and AE rats in both sexes, and Dual rats from MIA rats in females. These data suggest that there is information in these circuits regarding MIA and AE, and that activity in these regions is disrupted by these environmental stressors. Since cortical-striatal and cortical-hippocampal circuits have been previously shown to control responding to drugs and drug cues [11–13], we hypothesize that dysregulation in these circuits may underlie the increased drinking we see in Dual rats, and that amelioration of these circuit disruptions might reduce alcohol consumption, which is the focus of our ongoing research.

In the current study, MIA paired with AE was necessary to increase alcohol consumption in adulthood. While others have shown that AE alone increases alcohol drinking [29, 30], the results are inconsistent [31–33] and likely depend on the exposure time-point and regimen (e.g., continuous vs. intermittent). Further, while there are data to suggest that MIA alone alters reward behaviors in rodents, this work has almost entirely focuses on motor responses to dopamine agonists in male offspring [34–36]. Since dopamine activity is both decreased and increased, depending on brain region, in MIA rats [37, 38], it is unclear if these previous data represent an “addiction” phenotype, or are simply reflective of the locomotor deficits induced by dopamine dysfunction. A more recent set of studies indicate that MIA leads to enhanced dopamine firing in the VTA in male offspring [38]. Increased VTA dopamine firing has been previously demonstrated in male alcohol-preferring (P) rats, and rodents experiencing alcohol withdrawal [39], suggesting that MIA might lead to VTA dopamine changes that enhance the risk of alcohol misuse, at least in males. However, the current study did not see any increases in alcohol drinking in rats exposed to MIA alone. We, therefore, hypothesize that AE in this study impacted neurodevelopment at a critical time-point that synergistically interacted with MIA to produce increased adulthood drinking. These data support the hypothesis that “two-hits,” one in very early life and one in adolescence, are necessary to produce a phenotype that is similar to the clinical presentation of co-occurring disorders [9, 10]. Our future studies aim to advance this line of work by testing whether MIA and/or AE rats are willing to work harder for alcohol and other drugs than control rats in an operant setting.

It is important to highlight that we observed significant sex differences in these studies that may help us better understand the complex sex differences in clinical presentations of co-occurring disorders. Schizophrenia is more common in men, especially earlier in life, and men with schizophrenia are more likely to have a co-occurring SUD [40]. On the other hand, rates of alcohol misuse are increasing among women, and women experience more psychiatric issues related to alcohol misuse than men [41, 42]. Our works aims to begin to disentangle the neurobiological contributions to these sex differences. For instance, we replicated our previous studies showing that female rats drink more alcohol in general compared to males [25, 28], and also add that females might be more susceptible to the impact of the Dual hit on drinking behavior. Further, cortical-striatal-hippocampal oscillations were not predictive of Dual vs. MIA rats in males, as it was in females. These data suggest that the AE might impact female brain development more than it does in males. This is interesting in light of data showing that AE makes female mice more sensitive to stress-induced negative affect compared to males [43], and that females are more sensitive to stress-induced relapse compared to males [44]. We therefore hypothesize that AE on top of MIA might lead to dysregulated HPA-axis activity in females [45], which may underlie the increased drinking observed in these studies and is a topic of our ongoing work.

The primary neural features that differentiated across groups also differed between sexes. Predictive models distinguished Dual rats from control rats, and Dual rats from AE rats, relatively well in both sexes. However, an interesting pattern emerged when we looked at the most predictive neural features for each sex. Cortical features, particularly from the IL, largely contained the most information in predicting females from every other group. In males, the predictive features were more mixed; though a lot of the information still came from cortical sites, hippocampal and striatal features, as well as cortical-hippocampal coherence, were also predictive of male Dual rats. These data help us identify which neural features to target to try to reduce alcohol misuse in future studies. For example, we have previously shown that deep brain stimulation to the NAc shell has the capacity to reduce alcohol consumption in high-drinking male rats [27]. These data are only a small portion of the literature suggesting that neuromodulation-based therapies can be efficacious for addiction and other neuropsychiatric disorders [46], but these studies have almost exclusively been done in male animals. In order to successfully initiate neuromodulation-based therapies that will be relevant to clinical populations, it is vital to understand “where” and “what” (e.g., striatal vs. cortical oscillations) we should be targeting, and whether these parameters may be different in males vs. females. The present study is a promising first step in the process of identifying neural features that may underly maladaptive behaviors, and our future work will test the capacity for cortical vs. striatal vs. hippocampal stimulation to reduce drinking in male and female Dual rats.

There are a few important limitations to consider. First, while MIA did significantly enhance circulating TNFα, it did not significantly increase IL6 levels two hours after injection. However, we did see reduced weight gain and eating behavior in dams exposed to polyI:C, which is consistent with other reports [22, 23]. Combined with the hypolocomotion we observed in male MIA offspring, we are confident that polyI:C induced MIA in our study. In future studies, collecting blood at different timepoints (e.g., 90 minutes) may help capture the previously observed increases in pro-inflammatory cytokines [47, 48]. We also cannot rule out the possibility that injection stress may have impacted our cytokine data in the dams, as acute stress has been shown to enhance circulating IL6 and TNFα levels [49, 50]. Secondly, littermates were used in the same groups due to the prenatal exposure time-point for polyI:C. We thus controlled for litter effects in our analyses and found no effect of the dam on adolescent and adulthood alcohol drinking. However, there were litter effects for locomotor response to novelty, which could have been influenced by previously observed MIA-induced changes in maternal care behavior [51]. Our future studies will therefore measure maternal care behaviors in MIA and control dams to be used as a covariate in analyses, and try to distribute pups from each dam across different experiments. Finally, we did not observe MIA-induced changes to anxiety behaviors measured in the open field, as others have seen previously [52]. However, our primary aim was to measure locomotor behavior and the data was collected in the dark. We thus hypothesize that a more targeted and thorough analysis of negative affective behaviors in MIA offspring is warranted in future studies.

## Conclusion

The current data helps us begin to understand the neurobiological underpinnings of the behavioral and cognitive deficits linked to prenatal exposure to infection and adolescent alcohol exposure [53, 54]. Our data also provide support for a novel heuristic neurodevelopmental model which we can use to study the biological basis of co-occurring mental illness and substance use. Although the current study is specifically focused on alcohol drinking, these results have significant implications for other addictive substances like cannabis and nicotine, which are two of the other most commonly used drugs by individuals with mental illness [1]. In other words, the Dual model in the present study may serve as a translationally relevant platform on which we can better study the neurobiological underpinnings of, and develop treatments for, co-occurring disorders. These studies and our future work will contribute to the larger goal of identifying how early environmental stressors change the brain in such a way as to predispose an individual to develop a mental illness and/or SUD, uncovering biological treatment targets for therapeutic development.

## Supplementary information

Supplemental Methods and Results

Supplemental Figure 1

Supplemental Figure 2

Supplemental Figure 3
