# Supplementary material for: Maternal immune activation and adolescent alcohol exposure increase alcohol drinking and disrupt cortical-striatal-hippocampal oscillations in adult offspring
Source: Transl Psychiatry. 2022 Jul 20;12:288. doi: 10.1038/s41398-022-02065-y (PMC9300672; doi:10.1038/s41398-022-02065-y)
Supplement: Supplementary file 1 — Supplemental Methods and Results [file 41398_2022_2065_MOESM1_ESM.docx]

**Supplementary Results**

Sucrose fade data: During the first phase of alcohol drinking training (5% sucrose only), there was a main effect of sex [*F*(1,68) = 47.20, *p* < 0.001, *n^2^p* = 0.41], with post-hoc tests revealing that females drank more sucrose overall compared to males (*p* < 0.05; Supplementary Figure 1). During sucrose fade (5% sucrose + 10% alcohol, then 2.5% sucrose + 10% alcohol), a repeated measures ANOVA revealed a significant effect of phase [*F*(1,68) = 55.53, *p* < 0.001, *n^2^p* = 0.45], sex [*F*(1,68) = 17.42, *p* < 0.001, *n^2^p* = .20], MIA [*F*(1,68) = 6.52, *p* = 0.01, *n^2^p* = .09], and AE [*F*(1,68) = 4.46, *p* = 0.04, *n^2^p* = 0.06] on alcohol consumed (g/kg). There was also a phase*AE interaction [*F*(1,68) = 5.93, *p* = 0.02, *n^2^p* = 0.08], and a phase*sex*AE interaction [*F*(1,68) = 4.15, *p* = 0.046, *n^2^p* = 0.06], with post-hoc tests revealing that both male and female Dual rats drank more alcohol during the second phase (2.5% sucrose + 10% alcohol) compared to all other groups (*p* < 0.05; n =8-11/group/sex from 12 dams; Supplementary Figure 2).

**Supplementary Figure Captions**

**Supplementary Figure 1.** Average sucrose consumed (g/kg) across the first 5 days of drinking training. Females overall drank more sucrose than males (*p* < 0.05).

**Supplementary Figure 2.** Average alcohol consumed (g/kg) during sucrose fade training: 5% sucrose + 10% alcohol for 5 days, then 2.5% sucrose + 10% alcohol for 5 days. Both male and female Dual rats drank more alcohol during the second phase of sucrose fade training (*p* < 0.05).

**Supplementary Figure 3.** **A**) Representative raw LFP data from each brain region (R = right hemisphere; L = left hemisphere; NAc = nucleus accumbens shell; CA1 = dorsal hippocampus; PL = prelimbic mPFC; IL = infralimbic mPFC). **B**) Representative power spectral density for the left IL. **C**) Representative coherence between the left IL and left CA1.
