# Supplementary figures and images for: Maternal immune activation and adolescent alcohol exposure increase alcohol drinking and disrupt cortical-striatal-hippocampal oscillations in adult offspring
Source: Transl Psychiatry. 2022 Jul 20;12:288. doi: 10.1038/s41398-022-02065-y (PMC9300672; doi:10.1038/s41398-022-02065-y)

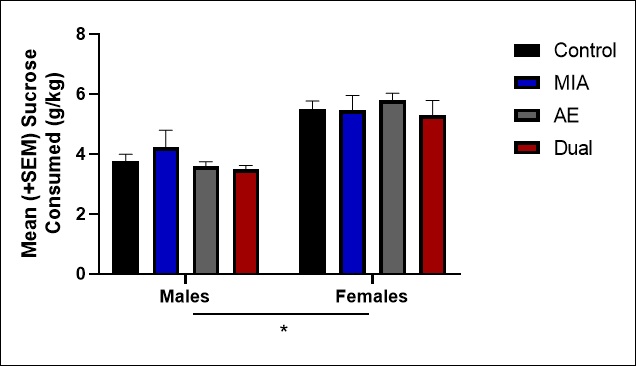

Supplement: Supplementary file 2 — Supplemental Figure 1 [file 41398_2022_2065_MOESM2_ESM.jpg]

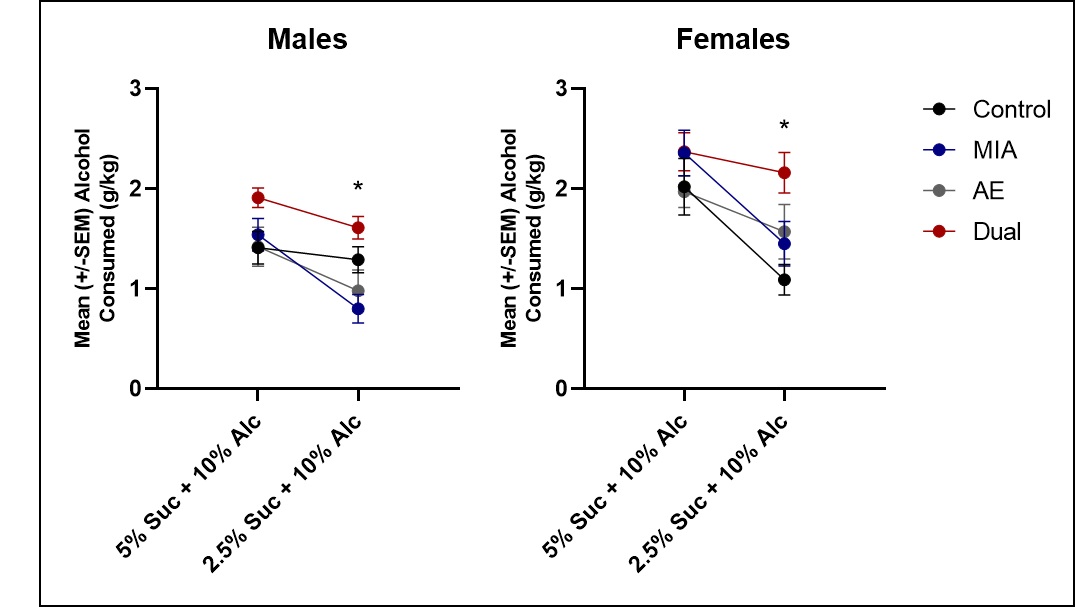

Supplement: Supplementary file 3 — Supplemental Figure 2 [file 41398_2022_2065_MOESM3_ESM.jpg]

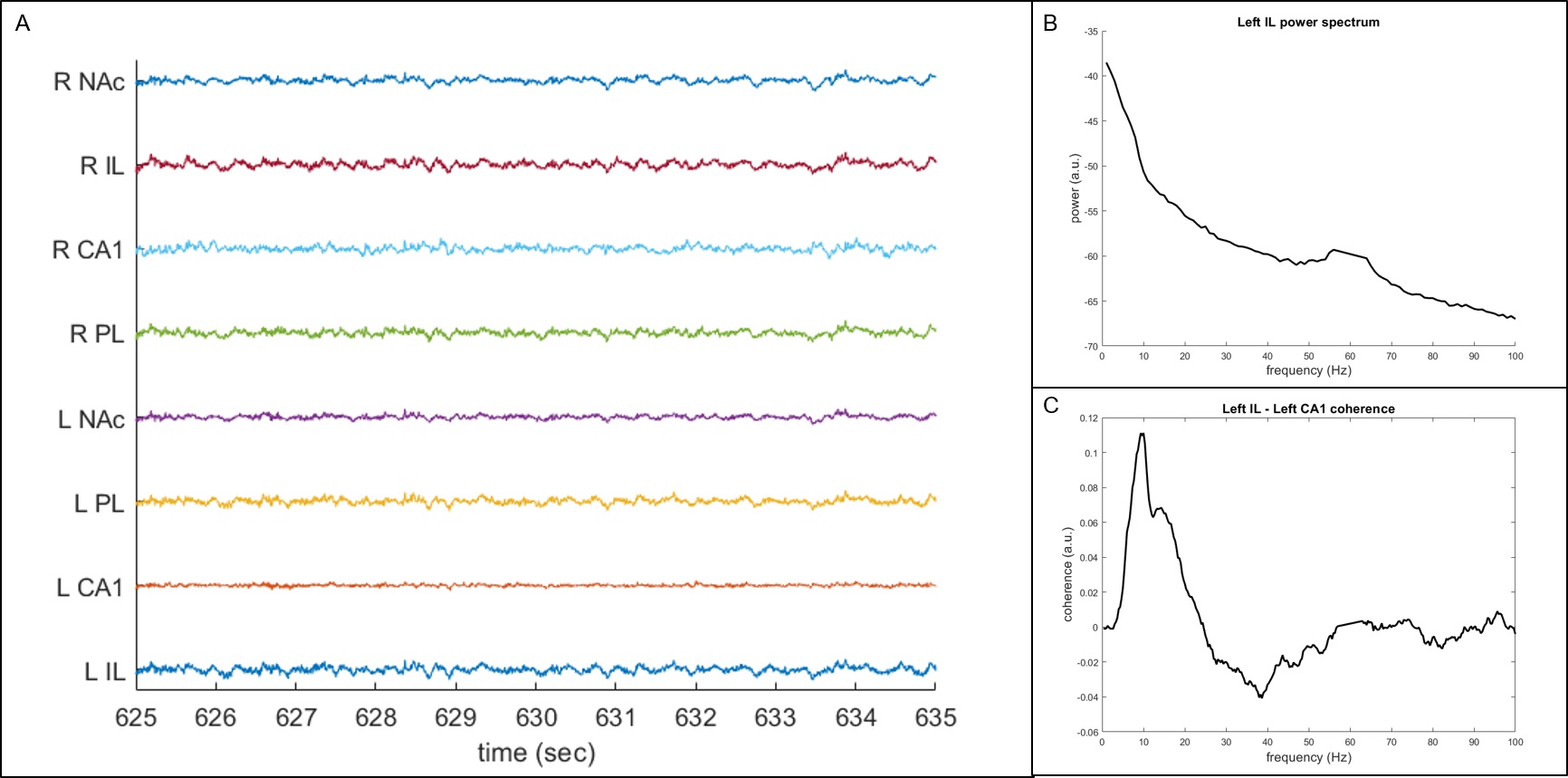

Supplement: Supplementary file 4 — Supplemental Figure 3 [file 41398_2022_2065_MOESM4_ESM.jpg]
